# Nutritional Enhancement and Quality Evaluation of Oat-Based Cookies Enriched with Doum Fruit (*Hyphaene thebaica* L.) Powder: A Comprehensive Assessment of Functional, Rheological, Physicochemical, Sensory, and Multivariate Characteristics

**DOI:** 10.3390/foods15193467

**Published:** 2026-09-28

**Authors:** Amal A. Matar, Alaa S. Alharbi, Maisa Bushra, Dina Ali, Nahed M. Rashed

**Affiliations:** 1 Food and Nutrition Sciences Department, College of Agricultural and Food Sciences, King Faisal University, Al-Ahsa 31982, Saudi Arabia; amatar@kfu.edu.sa (A.A.M.); asmalharbi@kfu.edu.sa (A.S.A.); mbahmed@kfu.edu.sa (M.B.); dali@kfu.edu.sa (D.A.); 2 Department of Arid Land Agriculture, College of Agricultural and Food Sciences, King Faisal University, Al-Ahsa 31982, Saudi Arabia

**Keywords:** doum fruit powder, *Hyphaene thebaica*, oat flour, gluten-free cookies, dietary fiber, antioxidant activity

## Abstract

The objective of this study was to explore the use of doum fruit powder (*Hyphaene thebaica* L.) as a functional ingredient in gluten-free oat-based cookies. Doum fruit powder (DFP) was used to replace oat flour at 5, 10, 15 and 20% levels, and the resulting cookies were analyzed for nutritional, antioxidant, physical, color and sensory characteristics. DFP had higher dietary fiber, mineral, phenolic compound and antioxidant activity content than oat flour. Increasing the DFP level significantly increased the crude fiber (5.81–8.69%), total phenolic content (37.08–50.04 mg GAE/100 g), antioxidant activity (15.67–24.11%) and mineral content, and decreased the caloric value. Higher substitution levels also produced darker cookies with lower volume and higher density. Although sensory scores decreased with increasing DFP levels, all formulations remained acceptable. Correlation analysis and principal component analysis revealed strong associations between fiber, phenolics, antioxidant activity, and mineral content, while indicating a trade-off between nutritional enhancement and sensory quality. Cookies with 5–10% DFP provided the best compromise between the improved nutritional value and the acceptability by consumers. The results proved that DFP can be successfully employed to produce functional gluten-free cookies with improved health-promoting properties.

## 1. Introduction

The growing prevalence of diet-related chronic disorders has intensified interest in developing cereal-based foods with improved nutritional quality and enhanced physiological benefits. Rather than relying on synthetic fortification, current research increasingly focuses on naturally derived ingredients capable of supplying dietary fiber, minerals, and antioxidant phytochemicals while maintaining product quality and consumer acceptability. Consequently, bakery products have become an attractive platform for the incorporation of plant-derived functional ingredients because of their widespread consumption and formulation flexibility [1,2,3,4]. Oat flour represents one of the most promising cereal ingredients for functional bakery applications because of its balanced nutritional profile and the presence of β-glucan, high-quality proteins, unsaturated lipids, vitamins, minerals, and diverse antioxidant compounds [5]. Regular consumption of oat-based products has been associated with beneficial effects on cardiovascular health, glycemic regulation, digestive function, and satiety [4,6,7,8,9]. Despite these nutritional and health-promoting attributes, further enrichment of oat-based products with complementary sources of dietary fiber and bioactive phytochemicals offers an opportunity to enhance their functional and nutritional value [6,7].

Doum palm (*Hyphaene thebaica* L.) is, a member of Arecaceae family, and is an indigenous fruit tree with a wide distribution in Egypt and other arid regions of Africa. The mesocarp of the doum is edible and traditionally fresh consumed, dried or as a drink. Previous studies have demonstrated that the doum fruit is rich in dietary fiber, minerals, flavonoids, and phenolic compounds other bioactive constituents with antioxidant activity. Moreover, potential health-promoting effects of doum fruit including antioxidant, antimicrobial, hypoglycemic and cardioprotective activities were found. The doum fruit powder due to its specific nutritional composition could be a promising functional ingredient to be added in bakery products [10,11]. Beyond its traditional use, doum fruit has attracted increasing interest as a functional food ingredient because of its dietary fiber, mineral content, phenolic compounds, and antioxidant potential. Previous studies have explored the incorporation of doum fruit or its powder into different food systems to improve their nutritional value and functional characteristics. Its incorporation has been associated with increased dietary fiber and mineral contents, as well as enhanced phenolic content and antioxidant activity, highlighting its potential as a plant-based ingredient for the development of value-added food products. In bakery applications, the use of doum-derived ingredients is particularly promising because they can contribute nutritional and bioactive compounds while providing an opportunity to diversify conventional cereal-based formulations. However, the extent to which these benefits can be achieved depends on the incorporation level and its potential effects on technological performance and sensory quality [12,13].

The use of natural sweeteners in place of refined sugar in the creation of healthier food products has also received a lot of attention. Date paste is a natural sweetener that enhances the nutritional profile of baked goods and is a desirable substitute for refined sugar because it is high in dietary fiber, minerals, and bioactive compounds [14].

Although oat flour and doum fruit powder have individually received attention for their nutritional and technological properties, their integration into oat-based cookie formulations provides an opportunity to develop bakery products with enhanced nutritional and bioactive value. The novelty of the present study lies in its comprehensive evaluation of the effect of graded doum fruit powder incorporation on the overall performance of oat-based cookies, spanning ingredient composition, dough rheology, nutritional and antioxidant properties, physical and color characteristics, sensory quality, and multivariate relationships. By integrating these complementary assessments, the study provides a broader understanding of the interactions between formulation composition, technological behavior, nutritional enhancement, and consumer acceptability and identifies a practical incorporation range for achieving a balanced product quality.

## 2. Materials and Methods

### 2.1. Materials

Doum fruits (*Hyphaene thebaica*), oat flour (*Avena sativa* L.), granulated sugar, shortening, skim milk powder, eggs, salt, baking powder, and vanilla flavor were purchased from a local market in Saudi Arabia. Date palm fruits (*Phoenix dactylifera* L. cv. Khalas) were used as a natural sweetening agent in cookie formulations. All chemicals, solvents, and reagents used for analytical determinations were of analytical grade.

### 2.2. Preparation of Doum Fruit Powder (DFP)

Doum fruits were washed with tap water containing sodium hypochlorite solution (0.05 g/L) (Sigma Chemical Co., St. Louis, MO, USA), completely cleaned with distilled water, then allowed to air dry. The fruits were manually cracked to separate the epicarp and edible mesocarp. The mesocarp was chopped into tiny pieces and dried for six hours at 60 °C in a hot-air oven, and milled using a laboratory grinder (Wiley Mill, Model 4, Thomas Scientific, Swedesboro, NJ, USA). The obtained powder was sieved through an 80-mesh sieve to obtain doum fruit powder (DFP), then stored in airtight glass containers at 5 °C until use [15].

### 2.3. Preparation of Date Paste

Date palm fruits (cv. Khalas) were manually deseeded, steeped in water for half an hour, and then blended into a homogeneous paste. In cookie recipes, the prepared date paste served as a natural sugar substitute.

### 2.4. Formulation, of Composite Flour Blends

Commercial oat flour labelled gluten-free by the manufacturer was used as the base cereal ingredient in this study. The product (Product/Barcode No. 614347235096) was obtained in its original commercial packaging, on which the gluten-free status was clearly indicated. Oat flour was partially substituted with DFP to create composite flour blends. levels of 0, 5, 10, 15, and 20% Table 1. These levels were selected to provide a progressive incorporation range for systematically evaluating the effects of increasing DFP addition on the nutritional, technological, physical, and sensory characteristics of the cookies and for identifying an appropriate balance between functional enhancement and product quality. Refined sugar was partially or completely replaced with date paste according to the experimental design. Two control treatments were prepared: Control 1 (C1), refined sugar, and Control 2 (C2), date paste as a sugar substitute. All dry ingredients were sieved through an 80-mesh sieve, thoroughly mixed to ensure homogeneity, and kept at 4 °C in sealed containers until needed.

### 2.5. Preparation of Cookies

Cookies were prepared according to the method described by Hooda & Jood [16] with slight modifications. A soft and fluffy texture was obtained by creaming shortening and sugar/date paste. After that, eggs and skim milk were added while being continuously mixed. To create a homogenous dough, flour mixtures, baking powder, vanilla, and salt were then progressively added. The dough was cut into circles with a diameter of 60 mm after being sheeted to a thickness of 6 mm. For fifteen minutes, baking was done at 180 °C. After cooling to room temperature, the cooked cookies were placed in airtight containers for additional examination Figure 1.

### 2.6. Chemical Composition Analysis

The proximate composition of doum fruit powder (DFP) and cookie samples, including moisture, crude protein, crude fat, crude fiber, and ash contents, was determined according to the official methods of the Association of Official Analytical Chemists [17], Moisture content was determined by oven drying at 105 °C to constant weight, crude protein by the Kjeldahl method using a nitrogen conversion factor of 6.25, crude fat by Soxhlet extraction with petroleum ether, ash by incineration in a muffle furnace at 550 °C, ash by incineration in a muffle furnace at 550 °C, and crude fiber according to [17]. Total carbohydrate content was calculated by difference as follows:

Total carbohydrates (%) = 100 − (moisture + crude protein + crude fat + ash + crude fiber).

The energy value was calculated using the general Atwater conversion factors (4 kcal g^−1^ for protein, 4 kcal g^−1^ for carbohydrates, and 9 kcal g^−1^ for fat) and expressed as kcal/100 g.

### 2.7. Determination of Mineral Content

A nitric acid–perchloric acid mixture (5:1, *v*/*v*) was used to wet digest around 0.5 g of dried sample in order to evaluate the mineral elements (Ca, Mg, K, Na, Fe, Zn, Cu, Mn, P, Ni, and Co). After filtering, the digested solution was diluted with deionized water to a predetermined level. An Atomic Absorption Spectrophotometer (AAnalyst 400, PerkinElmer Inc., Waltham, MA, USA) was used to measure calcium, magnesium, iron, zinc, copper, manganese, nickel, and cobalt. A flame photometer was used to measure potassium and sodium, while the molybdenum blue method was used to measure phosphorus colorimetrically. The units of measurement for mineral concentrations were mg/100 g dry weight [18].

### 2.8. Extraction of Bioactive Compounds

Bioactive compounds were extracted according to the method of [19] with slight modifications. In short, an orbital shaker (150 rpm) was used to extract 10 g of DFP and 100 mL of 80% ethanol for 72 h at room temperature. A rotary evaporator operating at 40 °C was used to concentrate the extract under low pressure after it had been filtered thru Whatman No. 1 filter paper. Before being examined further, the concentrated extract was kept at −20 °C.

### 2.9. Determination of Bioactive Compounds and Antioxidant Activity

Total phenolic content (TPC) was determined using the Folin–Ciocalteu method and expressed as mg gallic acid equivalents (GAE)/100 g dry weight [20]. Total flavonoid content (TFC) was measured using the aluminum chloride colorimetric assay and expressed as mg quercetin equivalents (QE)/100 g [21]. Total carotenoid content was determined spectrophotometrically according to Wellburn [22]. Antioxidant activity was evaluated using the DPPH radical scavenging assay described by Aryal et al. [21], and the results were expressed as percentage inhibition.

### 2.10. HPLC Analysis

An Agilent 1260 Infinity HPLC system with a quaternary pump, autosampler, diode-array detector, and Eclipse Plus C18 column (4.6 × 250 mm, 5 μm) was used to identify and quantify phenolic chemicals. A gradient elution of solvent A (0.2% phosphoric acid in water) and solvent B (methanol) at a flow rate of 1.0 mL min^−1^ was used for separation. The detection was done at 280 nm, and the injection volume was 20 μL. Individual phenolic compounds were identified by comparing their retention times and UV spectra with those of authentic standards [23].

### 2.11. Functional Properties of Doum Fruit Powder

Water absorption capacity (WAC), oil absorption capacity (OAC), water holding capacity (WHC), oil holding capacity (OHC), swelling capacity (SC), bulk density (BD), and emulsion capacity (EC) were determined according to the methods described by.

### 2.12. Rheological Properties of Dough

The rheological behavior of composite flour dough was evaluated using a Brabender Farinograph (Model Farinograph-E Brabender GmbH & Co. KG, Duisburg, Germany) according to AACC International Approved Method 54-21.02. Water absorption, dough development time, dough stability, mixing tolerance index, and degree of weakening were recorded automatically [24].

### 2.13. Physical Properties of Cookies

A digital Vernier caliper was used to measure the diameter and thickness of the cookies (±0.01 mm). The diameter to thickness ratio was used to compute the spread ratio. Cookie volume was determined by the rapeseed displacement method, and density was calculated as the ratio of cookie weight to volume [25].

### 2.14. Color Measurement

The color characteristics of the cookie samples were determined Using a colorimeter (CR-400, Konica Minolta, Chiyoda City, Japan) that was calibrated with a standard white plate (L* = 96.15, a* = 0.12, b* = 1.73), we measured the color difference (L*, a*, and b* values). The instrument was calculated according to the manufacturer’s instructions before measurement. The color parameters were expressed as lightness (L), redness/greenness (a), and yellowness/blueness (b). Measurements were taken at different positions on the surface of each cookie, and the mean values were used for statistical analysis. Each determination was performed in triplicate, and the results were expressed as mean ± standard deviation.

### 2.15. Sensory Evaluation

Sensory evaluation was performed by ten trained panelists from the Department of Food Science and Technology. Samples were coded with random three-digit numbers and presented in a randomized order under identical conditions. Appearance, color, odor, taste, texture, and overall acceptability were evaluated using a 10-point hedonic scale (1 = dislike extremely; 10 = like extremely). Water was provided for mouth rinsing between samples [26].

### 2.16. Statistical Analysis

All experimental determinations were performed in triplicate, and the results are expressed as mean ± standard deviation (SD). Statistical analyses were performed using IBM SPSS Statistics version 21.0 (IBM Corp., Armonk, NY, USA). One-way analysis of variance (ANOVA) was applied to evaluate the effect of different levels of doum fruit powder incorporation on the measured variables. When significant differences were detected, treatment means were separated using Duncan’s Multiple Range Test (DMRT) at *p* < 0.05.

To further investigate the relationships among the measured variables, Pearson’s correlation analysis was performed using standardized data to evaluate the strength and direction of linear associations among nutritional composition, bioactive compounds, antioxidant activity, mineral composition, physical characteristics, color attributes, and sensory properties. The correlation coefficients (r) ranged from −1 to +1, where positive values indicate direct relationships and negative values indicate inverse relationships.

Principal component analysis (PCA) was subsequently performed using standardized variables (zero mean and unit variance) to reduce data dimensionality, identify the major sources of variation, and visualize the relationships among cookie formulations and quality attributes. The first two principal components (PC1 and PC2), which explained the highest proportion of the total variance, were retained for interpretation and graphical presentation.

Pearson’s correlation matrix and PCA biplot were generated using Python (version 3.11) with the pandas, NumPy, scikit-learn, Matplotlib (version 3.11.2) and Seaborn (version 0.13.2) libraries.

## 3. Results

### 3.1. Proximate Composition of Oat Flour and Doum Fruit Powder

The proximate composition of oat flour (OF) and doum fruit powder (DFP) is presented in Table 2. Significant differences (*p* ≤ 0.05) were observed between the two raw materials. DFP contained significantly higher moisture, ash, crude fiber, and total carbohydrate contents than OF. In contrast, OF exhibited significantly higher crude protein, fat, and energy values. The crude fiber content of DFP (15.43 g/100 g) was approximately 57% higher than that of OF (9.84 g/100 g), whereas the ash content of DFP was nearly four times greater than that of OF, indicating its richness in mineral constituents. Conversely, the higher protein and lipid contents of OF contributed to its greater caloric value (393.37 kcal/100 g) compared with DFP (316.35 kcal/100 g).

### 3.2. Bioactive Compounds and Antioxidant Activity of Oat Flour and Doum Fruit Powder

The bioactive substances and antioxidant activity of the doum fruit powder (DFP) and oat flour (OF) are shown in Table 3. The two materials showed significant differences (*p* < 0.05). The total phenolic content of DFP (128.32 mg GAE/100 g) was substantially greater (*p* < 0.05) than that of OF (86.34 mg GAE/100 g). Additionally, DFP had far greater antioxidant activity (49.89%) than OF (32.48%). Conversely, OF had higher concentrations of β-carotene (12.32 mg/100 g) and total flavonoids (312.22 mg QE/100 g) than DFP (82.64 mg QE/100 g and 9.31 mg/100 g, respectively). These findings suggest that OF has larger quantities of flavonoids and β-carotene, while DFP has a higher phenolic content and antioxidant potential.

### 3.3. Mineral Composition of Oat Flour and Doum Fruit Powder

Mineral contents of oat flour (OF) and doum fruit powder (DFP) are shown in Table 4. The two materials showed significant differences. DFP was found to contain significantly higher levels of calcium (276.82 mg/100 g), magnesium (221.36 mg/100 g), potassium (2853.00 mg/100 g), sodium (258.48 mg/100 g) and iron (6.26 mg/100 g) compared to OF which contained 62.89, 134.12, 481.56, 42.11 and 3.08 mg/100 g, respectively. On the other hand, OF contained slightly higher levels of zinc (1.22 mg/100 g), copper (1.98 mg/100 g), manganese (4.78 mg/100 g) and phosphorus (401.56 mg/100 g) compared to DFP. Nickel and cobalt were only detected in DFP in trace amounts. Overall, potassium was the most abundant mineral in the two materials with DFP containing significantly higher levels compared to OF. Doum fruit powder exhibited markedly higher concentrations of potassium, calcium, magnesium, sodium, and iron than oat flour, confirming its potential as a natural source of dietary minerals and a promising ingredient for mineral enrichment of functional bakery products Figure 2.

### 3.4. Amino Acid Profile of Oat Flour and Doum Fruit

Figure 3 A,B shows the amino acid composition of oat flour (OF) and doum fruit powder (DFP). There were significant differences (*p* < 0.05) between the two raw materials. The OF had higher concentrations of all essential amino acids than DFP, except for methionine and cysteine which were similar in both raw materials. Leucine was the most abundant amino acid in OF (7.98 g/100 g protein) followed by valine (5.28 g/100 g protein), isoleucine (5.11 g/100 g protein) and lysine (4.43 g/100 g protein). DFP, on the other hand, contained much lower concentrations of most essential amino acids, with leucine (1.94 g/100 g protein) and histidine (1.61 g/100 g protein) being the most abundant. The total essential amino acids (EAA) reached 40.18 g/100 g protein in OF compared with 12.62 g/100 g protein in DFP. Similarly, total amino acids were markedly higher in OF (96.11 g/100 g protein) than in DFP (23.56 g/100 g protein). Furthermore, most essential amino acids in OF met or exceeded the FAO/WHO reference pattern for adults, whereas DFP showed lower values for several amino acids. The total essential amino acid content of oat flour was approximately 3.2 times higher than that of doum fruit powder, confirming the role of oat flour as the primary protein-contributing ingredient in the developed functional cookies.

### 3.5. HPLC Profiling of Phenolic Compounds

HPLC analysis showed different phenolic profiles for oat flour (OF) and doum fruit powder (DFP) (Table 5 and Figure 4). The major phenolic compound in oat flour was ferulic acid (162.12 mg kg^−1^), followed by p-coumaric acid (82.45 mg kg^−1^) and p-kaempferol (32.61 mg kg^−1^), as shown in Figure 4A, while other phenolic compounds were found in lower concentrations. Conversely, DFP presented a very different phenolic profile, with E-vanillic acid (366.27 mg kg^−1^) as the main phenolic compound followed by ellagic acid (220.44 mg kg^−1^), p-hydroxybenzoic acid (178.45 mg kg^−1^), epicatechin (112.56 mg kg^−1^) and protocatechuic acid (102.34 mg kg^−1^) (Figure 4B). In general, the total identified phenolic compounds in DFP (1352.82 mg kg^−1^) were approximately 3.5-fold higher compared to that of OF (387.53 mg kg^−1^), indicating the higher phenolic richness of doum fruit powder.

The Pareto analysis (Figure 4C) further demonstrated that a limited number of phenolic compounds contributed to the majority of the dominant phenolic fraction in DFP. In particular, E-vanillic acid, ellagic acid, p-hydroxybenzoic acid, epicatechin and protocatechuic acid accounted for almost 90% of the main phenolic compounds in the Pareto analysis while the other compounds contributed small proportions individually. The results showed that antioxidant activity of DFP is mainly due to a few major abundant phenolic compounds and not from the equal contribution of all identified compounds.

### 3.6. Functional Properties of Oat Flour and Doum Fruit Powder

The functional properties of oat flour (OF) and doum fruit powder (DFP) are presented in Table 6. Most functional properties showed significant differences (*p* ≤ 0.05) between the two raw materials. DFP had a significantly higher water absorption capacity (3.25 mL g^−1^) and oil holding capacity (0.98 g g^−1^) than OF suggesting its higher water and lipid retention ability. However, OF showed significantly higher oil absorption capacity (2.18 mL g^−1^), water holding capacity (2.09 g g^−1^), emulsion capacity (1.92%) and swelling capacity (5.32%) compared to DFP. Bulk density of the two materials did not differ significantly.

### 3.7. Comparative Nutritional and Functional Characteristics of Oat Flour and Doum Fruit Powder

Figure 5 presents a comparative overview of the nutritional and functional characteristics of oat flour (OF) and doum fruit powder (DFP). The radar plot revealed distinct compositional and functional profiles for the two ingredients. OF exhibited a higher protein content, whereas DFP showed significantly higher dietary fiber, ash, total phenolic content, antioxidant activity, water absorption capacity, and mineral contents, particularly potassium and calcium. DFP also exhibited higher total phenolic content and antioxidant activity than OF. Differences in the phenolic composition of the two ingredients were further observed by HPLC analysis, with DFP showing the presence of phenolic compounds, including ellagic acid, epicatechin, and hydroxybenzoic acid derivatives. In addition, DFP demonstrated a higher water absorption capacity than OF. Overall, Figure 5 highlights the distinct nutritional and functional characteristics of OF and DFP, with OF showing a higher protein content and DFP exhibiting higher levels of dietary fiber, minerals, phenolic compounds, antioxidant activity, and water absorption capacity.

### 3.8. Rheological Properties of Composite Flour Blends

The farinograph characteristics of oat flour substituted with different levels of doum fruit powder (DFP) are presented in Figure 6. Water absorption increased gradually with the increasing percentage of DFP from 64.40% in control to 71.45% in 20% DFP blend. Similarly, the dough development time increased gradually from 2.80 to 6.00 min, indicating that higher levels of DFP required longer mixing time to achieve optimum dough development. Moreover, the arrival time tended to increase with DFP incorporation, although some small fluctuations were observed at the intermediate substitution levels. In contrast, dough stability decreased continuously from 3.50 to 1.80 min, while both the mixing tolerance index and dough weakening increased markedly with increasing DFP level. Dough weakening increased from 148 BU in the control to 196 BU in the blend with 20% DFP indicating a continued decrease in dough resistance during mixing.

### 3.9. Chemical Composition, Bioactive Compounds, and Antioxidant Activity of DFP-Enriched Cookies

The addition of doum fruit powder (DFP) significantly (*p* ≤ 0.05) affected the chemical composition and functional quality of oat-based cookies (Table 7 and Figure 7). Significant increases in moisture, crude fiber, ash, total carbohydrate and total phenolic content and DPPH radical scavenging activity were observed with progressive increases in DFP substitution. The highest level of substitution (20% DFP) provided the cookies with the highest crude fiber (8.69%), ash (2.20%), total phenolics (50.04 mg GAE/100 g), and antioxidant activity (24.11%), which were approximately 50%, 72%, 35%, and 54% higher than the oat control (C2), respectively. Conversely, crude protein, crude fat, total flavonoids, β-carotene, and energy value decreased significantly as the proportion of DFP increased. The energy value decreased from 477.74 kcal/100 g in the oat control to 440.04 kcal/100 g at the level of substitution of 20%. Crude protein decreased from 17.81% to 13.76%. These results indicate that DFP enrichment improved the functional and antioxidant properties of the cookies and at the same time reduced their caloric density.

### 3.10. Mineral Composition of DFP-Enriched Cookies

The addition of doum fruit powder (DFP) significantly affected the mineral composition of oat-based cookies (Table 8 and Figure 8). All the measured minerals exhibited a progressive and significant (*p* ≤ 0.05) increase with the increase in the level of DFP substitution. The major mineral was potassium as shown in Figure 8A–E, with a significant increase from 284.22 mg/100 g in the control of the oat flour (C2) to 434.28 mg/100 g in the cookies with 20% DFP, which is an increase of approximately 52.8%. Phosphorus, magnesium, calcium and sodium showed similar increases, all reaching their maximum concentrations at the 20% substitution level. Calcium increased from 48.12 to 132.14 mg/100 g, while magnesium rose from 47.22 to 101.67 mg/100 g, representing approximately 2.7-fold and 2.2-fold increases, respectively. Likewise, sodium increased almost fourfold, from 35.83 to 142.11 mg/100 g, whereas phosphorus increased more moderately from 178.56 to 239.22 mg/100 g. The consistent upward trends shown in Figure 8 confirm that mineral enrichment was directly proportional to the level of DFP incorporated into the cookie formulation.

### 3.11. Physical and Color Characteristics of DFP-Enriched Cookies

The addition of doum fruit powder (DFP) significantly (*p* ≤ 0.05) affected oat-based cookies’ physical and color characteristics (Table 9 and Table 10; Figure 9). The increase in the substitution level of DFP produced a progressive reduction in the diameter and thickness of the cookies as shown in Figure 9A, while the spread ratio presented only minor variations along the formulations. Cookie diameter decreased from 64.37 mm in oat flour control (C2) to 56.63 mm at 20% DFP incorporation and thickness decreased from 7.67 to 6.58 mm. Although these changes in dimensions, the spread ratio did not change much except at the highest substitution levels where it showed a slight increase values at the 15% and 20% DFP substitution levels. The effect of DFP on the cookie structure can also be seen in Figure 9B, where the volume of cookies gradually decreased from 24.51 to 16.46 cm^3^, and the density significantly increased from 0.73 to 0.98 g cm^−3^. These results indicated that increasing DFP level resulted in cookies with more compact internal structure. Significant changes were also noted in cookie color (Table 9 and Figure 9C). The lightness (L*) and the yellowness (b*) decreased steadily as the DFP incorporation increased, whereas the redness (a*) increased significantly. The L* value decreased from 60.11 in the oat flour control to 50.58 at the highest DFP level, while a* increased from 6.32 to 7.34, indicating the development of a darker reddish-brown colour in the fortified cookies.

### 3.12. Sensory Evaluation of DFP-Enriched Cookies

The incorporation of doum fruit powder (DFP) significantly affected both the instrumental color characteristics and sensory properties of the oat-based cookies (Table 10; Figure 9C and Figure 10). Increasing the DFP substitution level produced progressive changes in the CIELAB color parameters, resulting in darker cookies, as indicated by the reduction in lightness (L) values. These instrumental changes were consistent with the sensory evaluation, where appearance and color scores decreased significantly (*p* ≤ 0.05) with increasing DFP incorporation. The sensory profile, presented in Figure 10A, was progressively modified as the DFP substitution level increased. All evaluated sensory attributes showed a gradual decrease compared with the control samples. However, cookies containing 5% DFP retained sensory scores close to those of the oat flour control (C2), indicating limited changes in consumer perception at the lowest substitution level. Similar declining trends were observed for odor, taste, and texture, with the greatest reductions generally recorded at the highest DFP incorporation level (20%). Despite the progressive decrease in sensory scores, all formulations remained within the acceptable sensory range, with mean scores exceeding 6.5 on the 10-point hedonic scale. As illustrated in Figure 10B, overall acceptability decreased gradually from 9.04 for the wheat control (C1) and 8.69 for the oat flour control (C2) to 7.09 for cookies containing 20% DFP. The formulation containing 5% DFP achieved a high overall acceptability score (8.24), while the formulations containing 10–20% DFP also remained sensorially acceptable.

### 3.13. Correlation Analysis and Principal Component Analysis

Pearson’s correlation analysis demonstrated strong relationships between the nutritional, physical, and sensory characteristics of DFP-enriched cookies (Figure 11A). There were very strong positive correlations (r = 0.94–0.99) among dietary fiber, total phenolics, antioxidant activity, potassium content, and density, which indicated that the increasing incorporation of DFP improved the nutritional and functional quality of the cookies at the same time. Conversely, these variables were highly negatively correlated with cookie volume, lightness (L*) and overall acceptability (r = −0.91 to −1.00), indicating that increases in nutritional value were associated with decreases in product expansion, brightness and sensory preference.

Cookie volume showed a strong positive correlation with overall acceptability (r = 0.99), suggesting that formulations with greater expansion and lower density were preferred by the sensory panel. Similarly, L* values were positively associated with overall acceptability (r = 0.92), indicating that lighter-colored cookies received higher sensory scores.

## 4. Discussion

The compositional differences between oat flour (OF) and doum fruit powder (DFP) demonstrate the complementary nutritional profile of the two ingredients. Higher protein and lipid contents of OF are consistent with the known nutritional composition of oat grains. Higher ash and crude fiber contents of DFP are in agreement with its high content in minerals and non-digestible carbohydrates [27,28,29]. DFP has lower energy value because of its lower fat content and higher dietary fiber fraction and can be used as an ingredient for the development of reduced-calorie bakery products. Such results suggest that the substitution of a portion of oat flour with DFP can increase the dietary fiber and mineral intake and reduce the caloric density of the final product simultaneously. Similar compositional characteristics have been reported for doum fruit and other fiber-rich plant materials incorporated into cereal-based foods [27,29,30]. Therefore, DFP is a functional ingredient of great potential for improving the nutritional value of oat flour and offering a practical approach to the development of healthier bakery products without the use of synthetic fortifying agents.

The divergence in bioactive profiles of OF and DFP suggests the nutritional complementarity of the two ingredients. The higher total phenolic content and antioxidant activity of DFP showed that doum fruit is a good source of natural phenolic antioxidants that can scavenge free radicals and reduce oxidative stress. Antioxidant activity is mainly due to high content of phenolic compounds that act through hydrogen atom donation and electron transfer mechanisms. Conversely, OF showed higher total flavonoids and β-carotene contents, in agreement with the typical phytochemical profile of oats, which are known as important sources of flavonoids and carotenoid pigments with antioxidant and provitamin A activities [29,31]. Thus, the combination of OF with DFP gives a complementary antioxidant protection, combining the phenolic-rich profile of DFP with the flavonoid- and carotenoid-rich composition of oats. This synergistic effect increases the functional value of the formulated cookies and supports the utilization of DFP as a natural ingredient for the development of antioxidant enriched bakery products [30,32].

The mineral profile showed that DFP is particularly rich in macroelements, mainly potassium, calcium, magnesium and sodium. OF exhibited relatively higher concentrations of phosphorus, manganese, copper and zinc [27,32,33]. Nutritionally, the predominance of potassium in DFP is important because of its role in electrolyte balance, nerve impulse transmission and cardiovascular function, while the elevated calcium and magnesium contents may contribute to bone health and normal metabolic activity. The higher iron content of DFP also improves its nutritional value, especially in cereal-based products where fortification with iron is desired. These results are in line with the higher ash content previously observed for DFP, confirming its mineral-rich composition [27,32]. Thus, the partial replacement of oat flour with DFP is an effective strategy to increase cookies’ mineral density, while improving their overall nutritional quality, supporting the development of naturally fortified bakery products [25].

The amino acid profile showed that the protein quality of OF was higher than DFP because most of the essential amino acids (especially leucine) were present in OF at higher concentrations. Leucine is known to be important in protein synthesis and muscle metabolism [34,35,36]. These results agree with the well-documented nutritional properties of oat proteins and confirm the prevalence of OF as a protein source in the formulated products. DFP had relatively higher levels of sulfur-containing amino acids such as methionine and cysteine, although the levels of most amino acids were lower. This may partly complement the balance of amino acids of cereal proteins [34,35,37]. The lower amino acid content is not likely to affect the nutritional quality of the composite flour since DFP was mainly developed to improve dietary fiber, mineral content and antioxidant properties and not to enrich protein. Thus, the combination of OF with DFP results in a balanced formulation that maintains the good protein quality of oats, and at the same time improves the functional and nutritional characteristics of the final bakery product [36,37,38,39,40].

HPLC analysis showed significant differences in phenolic composition of OF and DFP confirming more phytochemical diversity of DFP. The major phenolic in OF was Ferulic acid which consisted of approximately 42% of the identified phenolics followed by p-coumaric acid. This is in agreement with previous reports that cereals are important sources of hydroxycinnamic acids [28,41,42]. In contrast, DFP showed a significantly higher phenolic profile, with the total identified phenolic concentration being approximately 3.5-fold higher than that of OF. The presence of E-vanillic acid, ellagic acid and p-hydroxybenzoic acid in high amounts confirms that these compounds are the main constituents in the phytochemical composition of doum fruit. Epicatechin and protocatechuic acid are present, which further points to the variety of bioactive constituents naturally found in DFP, which have been widely reported to have antioxidant and health-promoting effects [43,44,45,46]. These results indicate that DFP is a good source of natural phenolic compounds and confirm its use as a functional ingredient to improve the phytochemical quality of bakery products based on oats.

The functional properties of DFP make it a suitable bakery ingredient that could be employed to modify the dough behavior and improve the product quality. The markedly higher water absorption and oil holding capacities of DFP are attributed to its greater dietary fiber content and porous structure, which provide a large number of hydrophilic and lipophilic binding sites for water and oil respectively [47,48,49]. These properties are desirable for moisture retention, flavor retention and delayed stalling in storage. However, OF had greater oil absorption, swelling and emulsifying capacities, mainly due to its higher starch, β-glucan and protein contents, which contribute to hydration and interfacial stabilization [49,50]. The differences in bulk density were not significant, suggesting that the incorporation of DFP will not affect any powder handling or mixing properties. Altogether, these functional properties indicate that DFP can be successfully incorporated in oat-based bakery formulations to improve water retention and technological performance, while providing a suitable base for subsequent dough processing and baking [51,52,53]. The progressive color changes observed with increasing DFP incorporation are consistent with the distinct color characteristics and compositional properties of doum fruit powder. The reduction in L values indicates a darker appearance as the proportion of DFP increased, which became particularly evident at the higher substitution levels. Such changes in color are technologically relevant because visual appearance is one of the first quality attributes perceived by consumers and may therefore influence product acceptance. In the present study, the progressive modification of cookie color was accompanied by a decline in sensory color scores at higher DFP levels, suggesting that excessive incorporation may negatively affect consumer perception. Nevertheless, moderate DFP incorporation, particularly at 5–10%, maintained relatively favorable sensory acceptance while providing nutritional and functional improvements.

The lack of significant differences in bulk density indicates that the replacement of oat flour with DFP is not likely to significantly affect the packing characteristics of the powder. In conclusion, the functional properties of DFP indicate its potential application as a functional ingredient in bakery products especially for the improvement of water absorption and oil retention characteristics Farinograph analysis showed that DFP had a significant effect on dough rheology that increased water absorption, dough development time and decreased dough stability. The higher water absorption is consistent with the functional properties of DFP discussed earlier and indicates the higher affinity of its dietary fiber to water [54,55]. The increased dough development time indicates that more mixing was needed to reach complete hydration and dough formation, as is common in fiber-enriched cereal formulations [56,57,58]. However, the reduction in dough stability and the increase in mixing tolerance index and dough weakening shows that DFP gradually broke the continuity of the dough matrix by diluting the protein network and absorbing insoluble fibrous particles [58,59]. However, the reduction in dough stability and the increase in mixing tolerance index and dough weakening shows that DFP gradually broke the continuity of the dough matrix by diluting the protein network and absorbing insoluble fibrous particles.

The addition of DFP significantly improved the nutritional quality of oat cookies by increasing crude fiber, ash, total phenolic content and antioxidant activity, and reducing fat content and caloric value. The incremental increase in fiber and ash is a clear sign of the successful transfer of dietary fiber and mineral constituents from DFP to the final product whereas the elevated moisture content is consistent with the improved water-binding properties of the composite flour [60,61]. The enrichment of phenolic compounds in the cookies was associated with a proportional increase in antioxidant activity, which suggests that the bioactive constituents of DFP remained stable during the baking process and continued to contribute to the functional quality of the product [57,58]. Rather, the reductions in protein, crude fat and energy value are mainly due to the differences in the composition of OF and DFP [59,61,62]. Generally, these findings suggest that DFP can be successfully incorporated into oat-based cookies to obtain nutritionally enriched products with higher antioxidant potential and lower energy density, without negatively affecting their technological suitability.

The increasing mineral content of oat-based cookies with increasing DFP incorporation confirms the effective enrichment of the final product with essential macroelements. All minerals (potassium, calcium, magnesium, sodium and phosphorus) were significantly increased, indicating the high mineral content of DFP and the retention efficiency during baking [32,63,64]. The greatest increase was shown by potassium, which shows that DFP can be an excellent natural source of this vital electrolyte in cereal-based products. The fortified cookies are enriched by the simultaneous increase in calcium and magnesium, which further increases the nutritional value of the fortified cookies and their contribution to bone health and normal physiological functions [65,66]. These results show that DFP is a viable alternative to fortification with synthetic minerals and maintains the technological suitability of the product. Therefore, the addition of DFP not only improved the functional quality of oat-based cookies but also enhanced their micronutrient density which encouraged the production of bakery products with enhanced nutritional value [29,66,67,68]. The physical properties of oat-based cookies were significantly affected by the DFP addition, with progressive reduction in diameter, thickness and volume and increase in density. The changes are consistent with the rheological properties of the composite dough, as reduced dough stability and increased water uptake restricted the dough expansion during baking [32,64]. The small variations in spread ratio suggest that DFP affected the expansion of cookies more than the flow of dough, indicating that structural changes during baking were mainly related to changes in the internal dough matrix rather than excessive spreading. The increase of cookie density further confirms the formation of a more compact structure with the increase of substitution level. [66,67,68]. The instrumental color measurements showed a steady decrease in the L* and b* values and an increase in the a* values, which suggested the production of darker and more reddish cookies with the increasing incorporation of DFP. The color changes observed are explained by the natural pigments of the doum fruit, and the enhanced Maillard reaction and caramelization during baking, which are common in fiber-rich bakery formulations [69,70]. The physical integrity of the cookies was preserved in all formulations, indicating that DFP can be successfully incorporated into oat-based cookies, contributing to the improved nutritional value with acceptable technological quality [71].

Sensory evaluation showed progressive reductions in the scores of appearances, color, odor, taste, texture, and overall acceptability with increasing levels of DFP, but all the formulations were within the acceptable range. The decrease in sensory scores is the combined effect of physical and color changes in the fortified cookies and not the effect of a specific quality attribute. The sensory attributes of cookies containing 5% DFP were like those of the oat flour control, suggesting that low levels of substitution had little impact on consumer perception. Higher DFP levels led to lower preference scores, but overall acceptability scores were above 7.0 for all formulations indicating good consumer acceptance. These findings demonstrate that DFP can be incorporated into oat-based cookies at moderate substitution levels to enhance nutritional functionality while maintaining the sensory quality required for consumer acceptance, supporting its potential application in the development of clean-label functional bakery products [29,70].

Pearson’s correlation analysis and PCA provided an integrated interpretation of the relationships among the nutritional, physical, color, and sensory characteristics of DFP-enriched cookies. The strong positive correlations among dietary fiber, mineral content, total phenolics, antioxidant activity, and density indicate that these attributes were simultaneously enhanced as the level of DFP increased. In contrast, these variables were negatively associated with cookie volume, lightness (L*), and overall acceptability, demonstrating the trade-off between nutritional enhancement and certain technological and sensory characteristics. These relationships confirm that the compositional changes introduced by DFP directly influenced the structural and sensory properties of the final product.

The PCA biplot further differentiated the cookie formulations according to their overall quality attributes. The highest DFP substitution level was closely associated with improved nutritional and functional properties, whereas the control formulation was characterized by superior physical and sensory quality. Intermediate formulations occupied transitional positions between these two extremes, indicating a gradual shift from sensory-oriented to nutrition-oriented characteristics as DFP incorporation increased. Among the tested formulations, the 10% DFP treatment exhibited the most balanced distribution within the PCA space, reflecting a favorable compromise between enhanced nutritional value and acceptable technological and sensory performance. Therefore, multivariate analysis confirmed that moderate DFP incorporation represents the optimum strategy for producing nutritionally improved oat-based cookies without markedly reducing product quality.

The selection of the 10% DFP formulation as the most desirable treatment was based on the overall balance among nutritional improvement, functional enhancement, technological properties, and sensory acceptability rather than on the maximization of a single attribute. At this incorporation level, the cookies showed clear improvements in crude fiber, mineral content, total phenolic content, and antioxidant activity compared with the oat control, while maintaining acceptable physical characteristics and relatively high sensory scores. Although further increases in DFP to 15 and 20% resulted in greater enrichment in fiber, minerals, phenolic compounds, and antioxidant activity, these higher substitution levels were accompanied by more pronounced changes in cookie weight, diameter, thickness, volume, density, and color, together with a progressive decline in sensory scores, particularly for taste, texture, and overall acceptability. Therefore, the 10% DFP level represented a practical compromise between nutritional enhancement and product quality. Importantly, the 5% formulation maintained sensory characteristics closer to those of the controls, whereas the 10% formulation provided a greater nutritional and antioxidant improvement without the more substantial deterioration in physical and sensory quality observed at 15 and 20% DFP. This distinction supports the selection of 10% DFP as the most balanced formulation, while the 5% level may be considered preferable when minimizing changes in sensory characteristics is the primary objective. Thus, the optimal incorporation level depends on the intended product priority; however, when both functional enhancement and consumer acceptability are considered simultaneously, the 10% DFP formulation provides the most favorable overall balance.

## 5. Conclusions

This study revealed that doum fruit powder (DFP) is a promising natural functional ingredient for the development of nutritionally enriched cookies based on oat. The addition of DFP significantly enhanced the nutritional value of cookies by improving dietary fiber, mineral content, total phenolic compounds and antioxidant activity and reducing fat content and energy value. DFP affected also the dough rheology and the physical properties of the final product, resulting in denser cookies with darker color as the substitution level increased. All formulations were sensorially acceptable even with these technological changes, showing overall acceptability scores above 7.0 on the 10-point hedonic scale. Multivariate statistical analyses further demonstrated a clear relationship between nutritional enhancement and technological performance. PCA showed that the 10% DFP formulation was the best treatment in terms of compromise between enhancement of nutritional and functional properties and acceptable physical and sensorial quality. The results showed that DFP could be used as a natural additive to improve the nutritional value of the bakery products without any artificial fortification. Overall, the present study provides a comprehensive evaluation of the compositional, functional, rheological, physicochemical, sensory, and multivariate characteristics of DFP-enriched oat cookies, offering valuable scientific evidence for the utilization of doum fruit as a sustainable functional food ingredient. Future studies should investigate the shelf life, bioaccessibility of phenolic compounds, and consumer acceptance under commercial production conditions to further support industrial application.

## Figures and Tables

**Figure 1:**
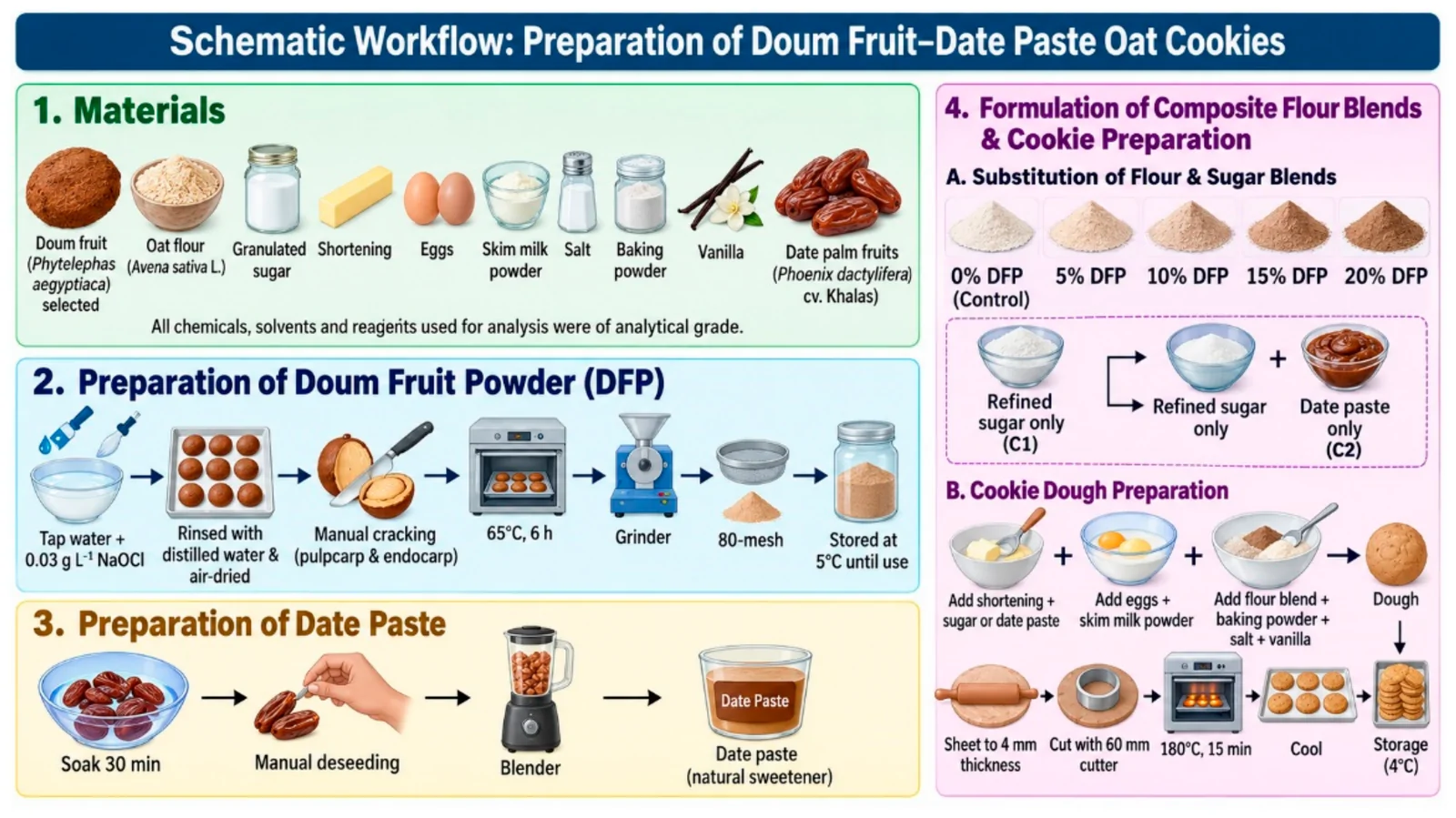
Schematic Workflow: preparation of oat cookies fortified with doum fruit powder. The figure was initially generated using FigureLabs v1.0(AI-assisted figure generation) and subsequently reviewed, scientifically verified, and manually edited by the authors to improve accuracy, layout, and compliance with the journal guidelines.

**Figure 2:**
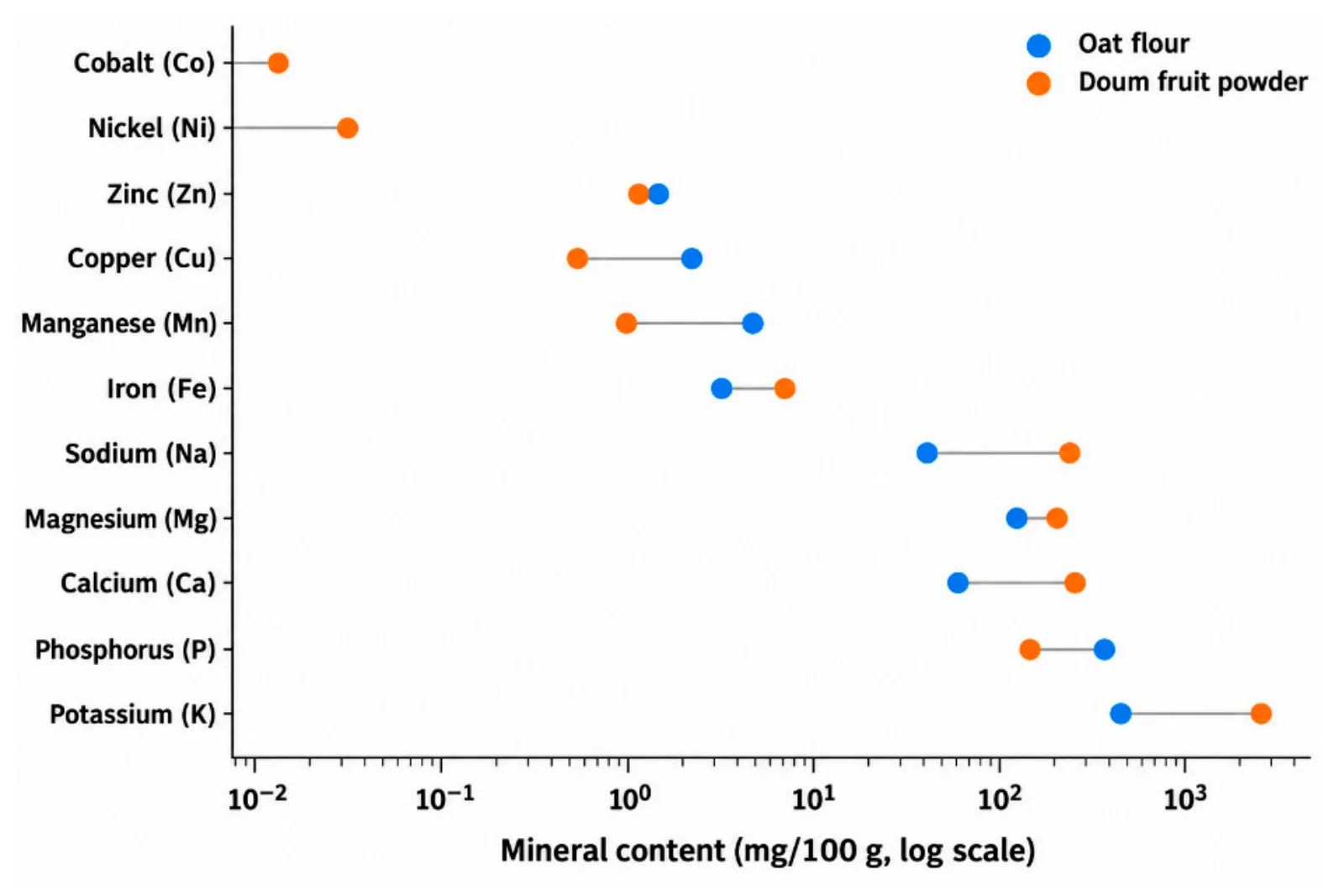
Comparison of the mineral composition of oat flour (OF) and doum fruit powder (DFP). Values are expressed as mean ± SD (*n* = 3). The logarithmic scale was used to facilitate comparison between macro- and trace minerals. Error bars represent the standard deviation.

**Figure 3:**
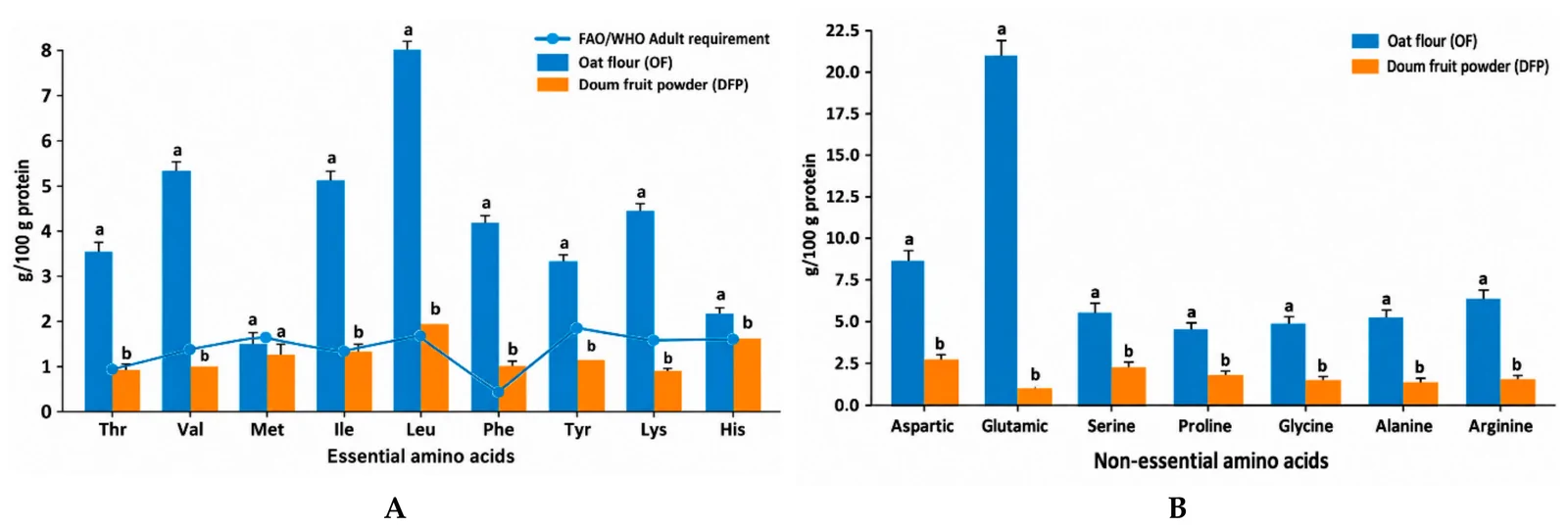
Essential amino acid profile of oat flour (OF) and doum fruit powder (DFP) compared with the FAO/WHO adult amino acid requirement pattern (**A**) and Non-essential amino acid profile of oat flour and doum fruit powder (**B**). Different lowercase letters indicate significant differences between means according to Duncan’s multiple range test at *p* ≤ 0.05.

**Figure 4:**
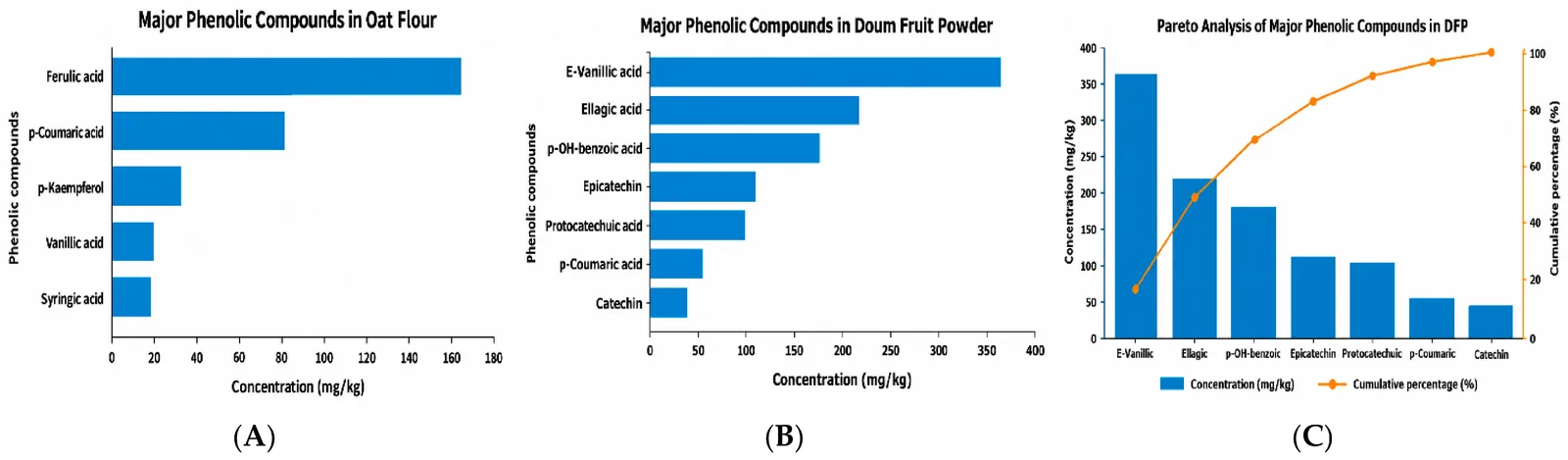
HPLC profile of the major phenolic compounds identified in oat flour (OF) and doum fruit powder (DFP). (**A**) Major phenolic compounds in OF, (**B**) major phenolic compounds in DFP, and (**C**) Pareto analysis showing the cumulative contribution of the dominant phenolic compounds in DFP. In panel C, bars indicate the concentration of individual phenolic compounds (mg/kg), while the line indicates the cumulative percentage of the total phenolic concentration accounted for by the compounds in descending order of concentration.

**Figure 5:**
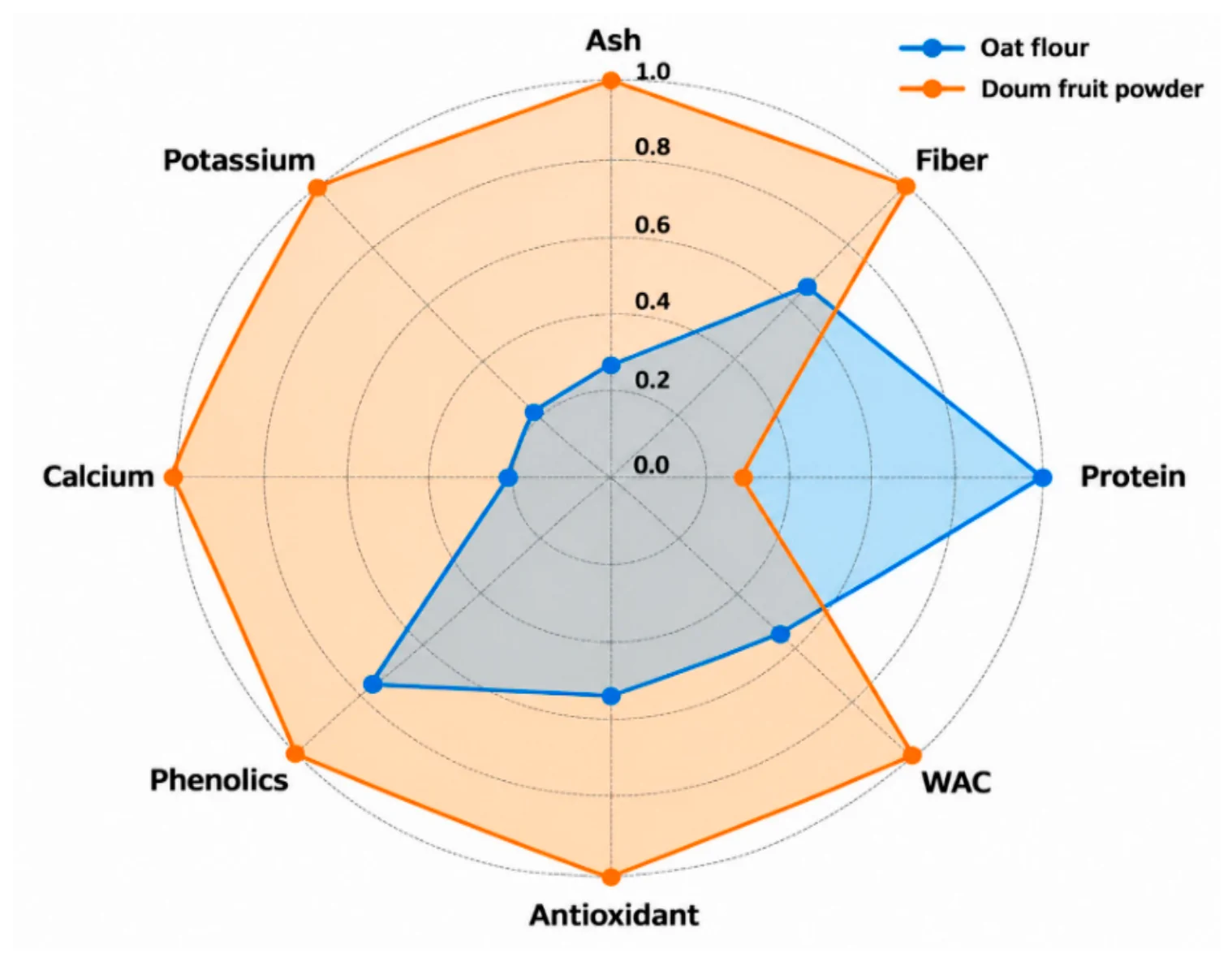
Comparative nutritional and functional characteristics of oat flour and doum fruit powder. Values were normalized relative to the maximum value of each parameter.

**Figure 6:**
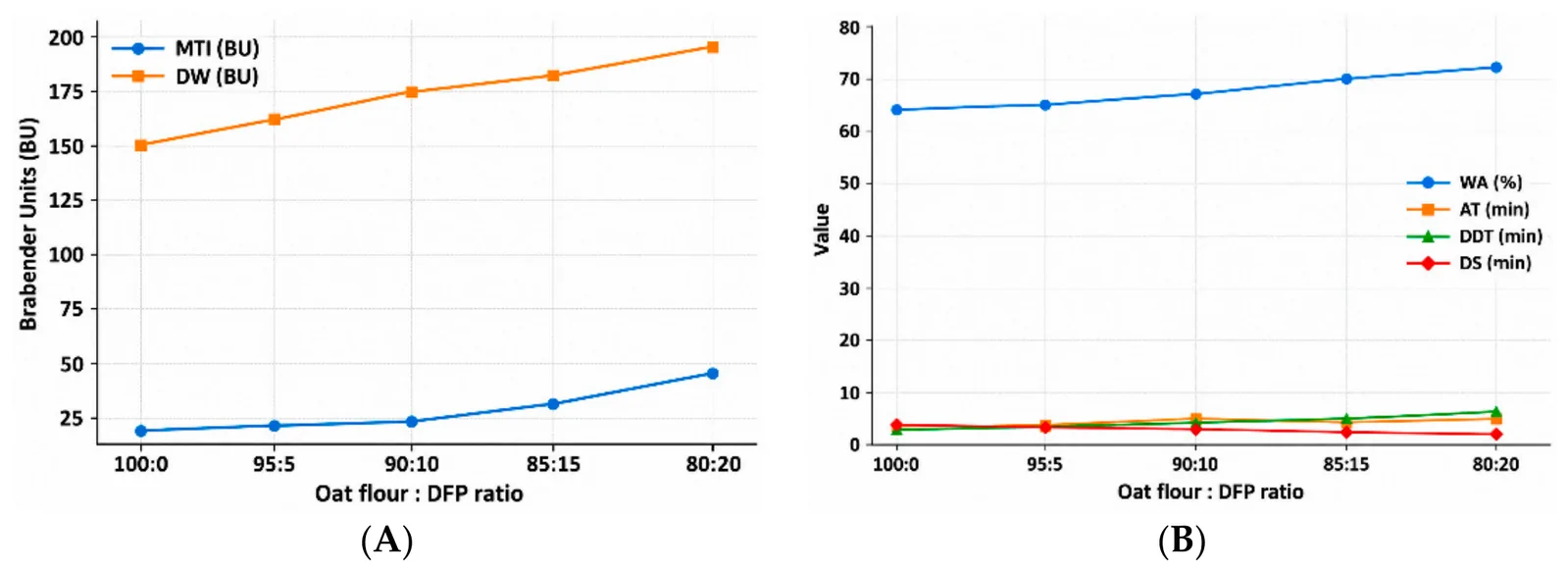
Effect of increasing doum fruit powder (DFP) substitution on the farinograph characteristics of oat flour blends. (**A**) Water absorption (WA), arrival time (AT), dough development time (DDT), and dough stability (DS). (**B**) Mixing tolerance index (MTI) and dough weakening (DW).

**Figure 7:**
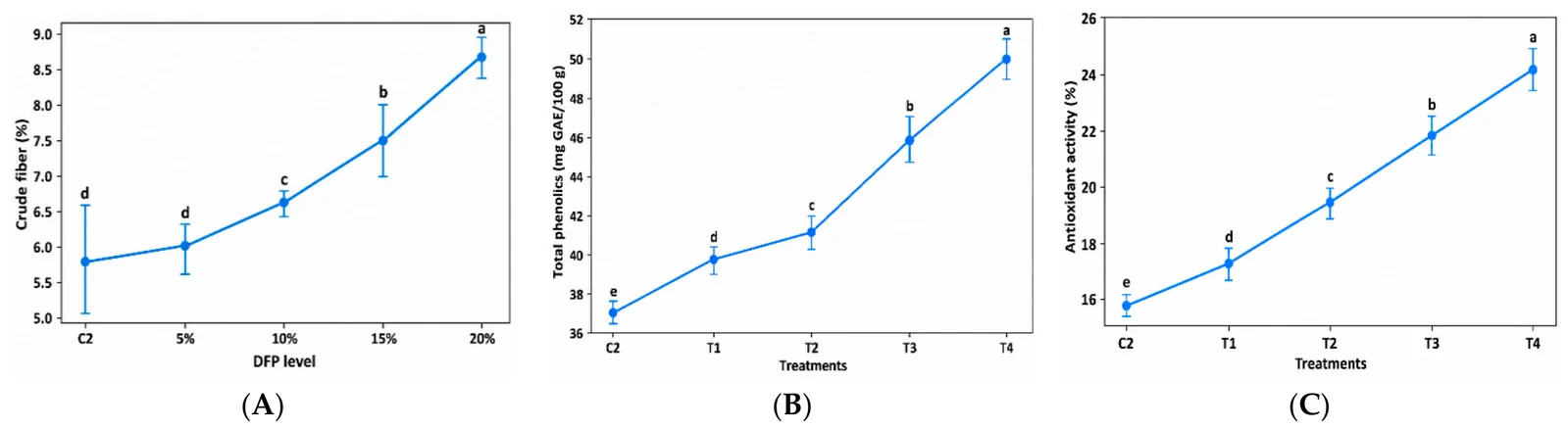
Effect of doum fruit powder (DFP) incorporation on selected nutritional and functional attributes of gluten-free cookies. (**A**) Crude fiber, (**B**) total phenolic content, and (**C**) antioxidant activity. Error bars represent standard deviation (*n* = 3). Different letters indicate significant differences among treatments at *p* ≤ 0.05 according to Duncan’s multiple range test.

**Figure 8:**
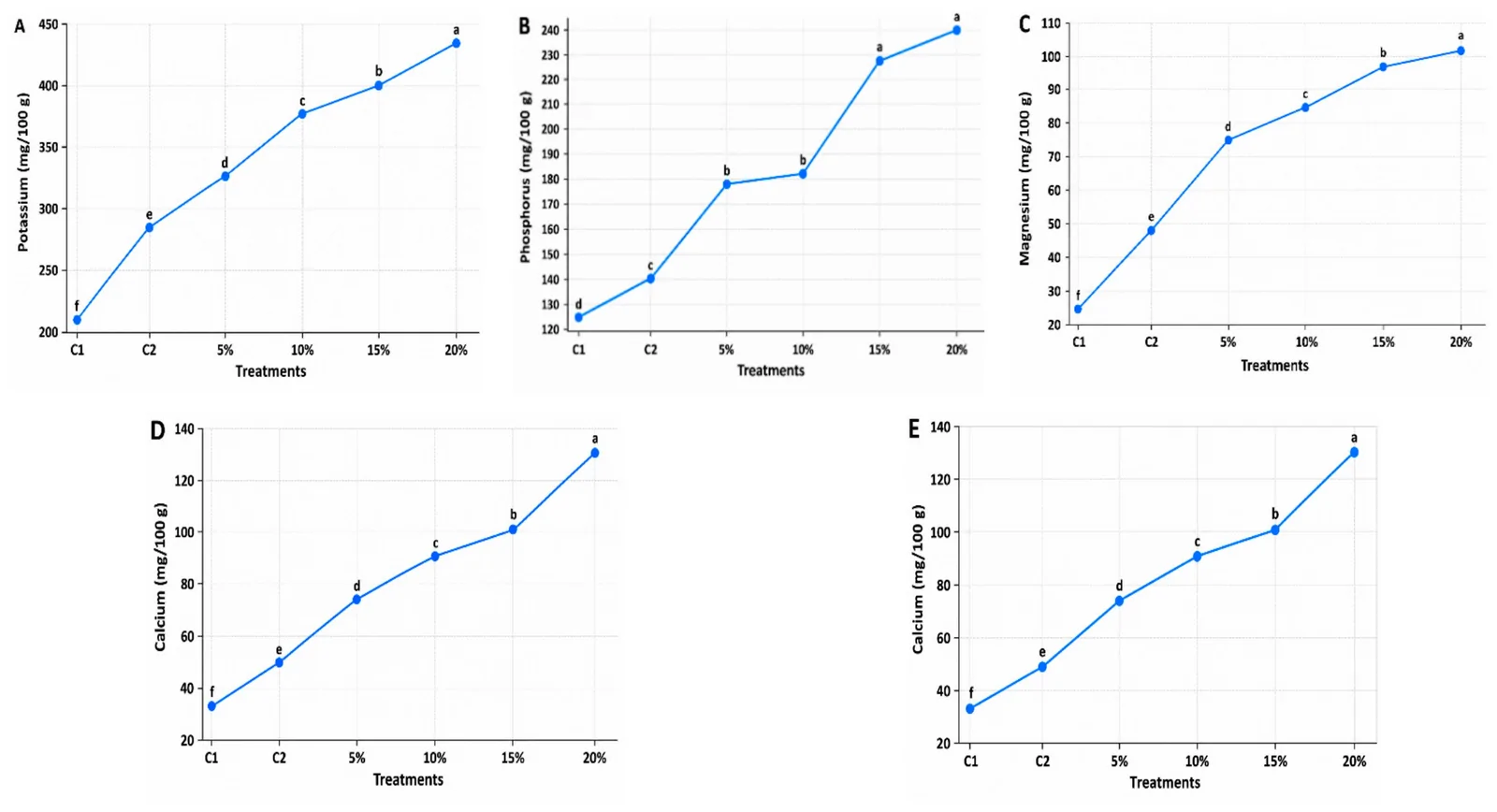
Effect of doum fruit powder (DFP) incorporation on the mineral composition of oat-based cookies: (**A**) potassium, (**B**) phosphorus, (**C**) magnesium, (**D**) calcium, and (**E**) sodium. Values are expressed as mean ± SD (n = 3). Error bars represent the standard deviation. Different letters indicate significant differences among treatments according to Duncan’s multiple range test (*p* ≤ 0.05).

**Figure 9:**
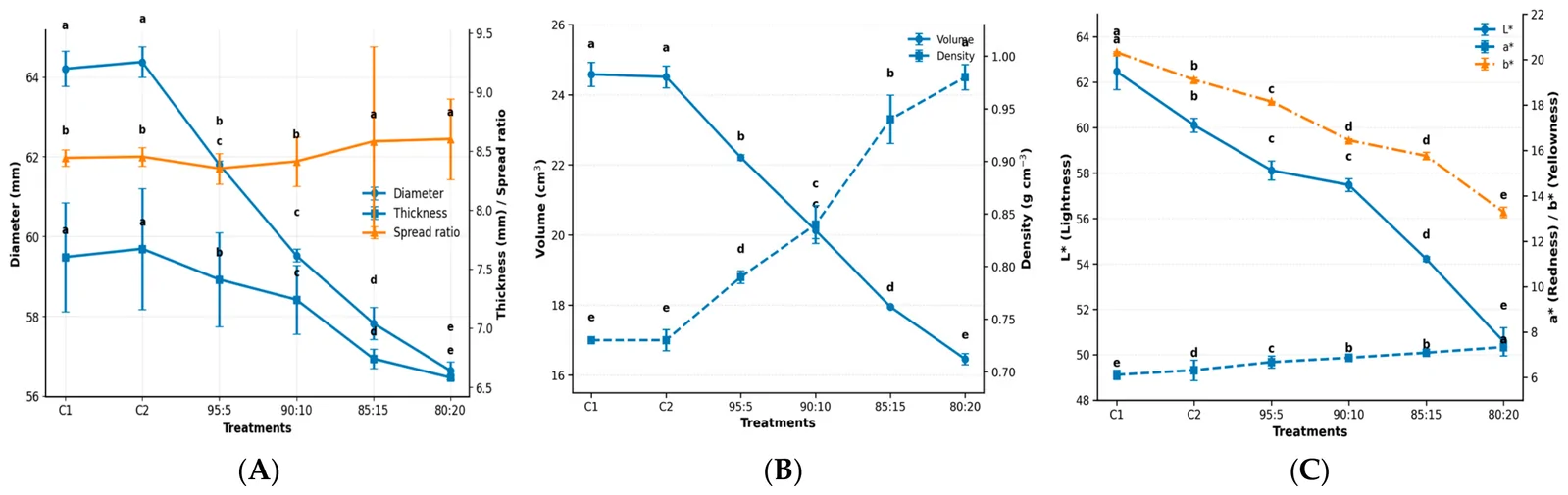
Changes in the physical and color properties of oat-based cookies as affected by increasing levels of doum fruit powder (DFP): (**A**) cookie dimensions (diameter, thickness, and spread ratio), (**B**) volume and density, and (**C**) CIE color attributes (L*, a*, and b*). Different lowercase letters indicate significant differences among treatment means for the corresponding attribute according to Duncan’s multiple range test at *p* ≤ 0.05.

**Figure 10:**
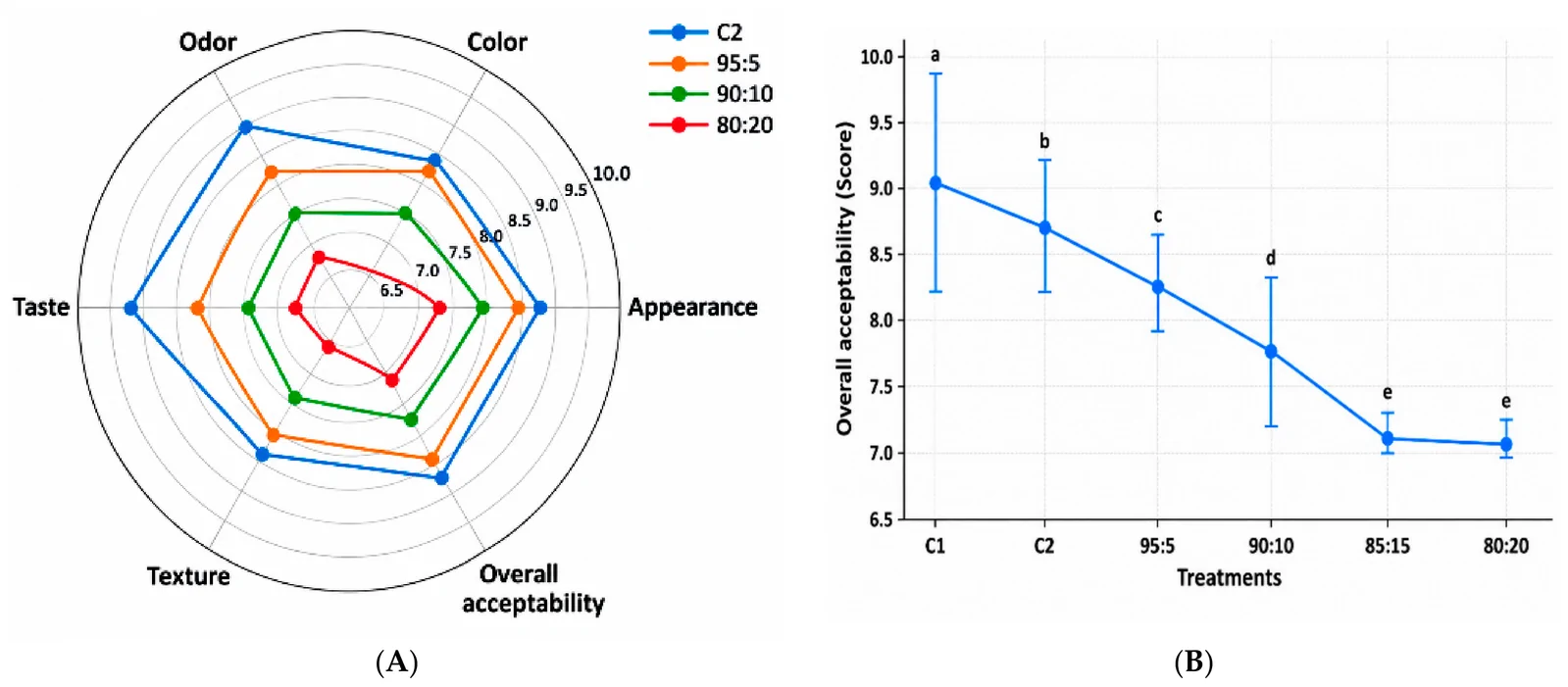
Sensory quality attributes of oat-based cookies are fortified with different levels of doum fruit powder (DFP). (**A**) Radar chart illustrates the sensory profile of cookies, including appearance, color, odor, taste, texture, and overall acceptability as affected by increasing levels of DFP incorporation. (**B**) Overall acceptability scores of oat-based cookies formulated with different levels of DFP. Values are expressed as mean ± standard deviation (SD) (*n* = 10). Error bars represent standard deviations. Different lowercase letters indicate significant differences among treatments according to Duncan’s Multiple Range Test (DMRT) at *p* ≤ 0.05.

**Figure 11:**
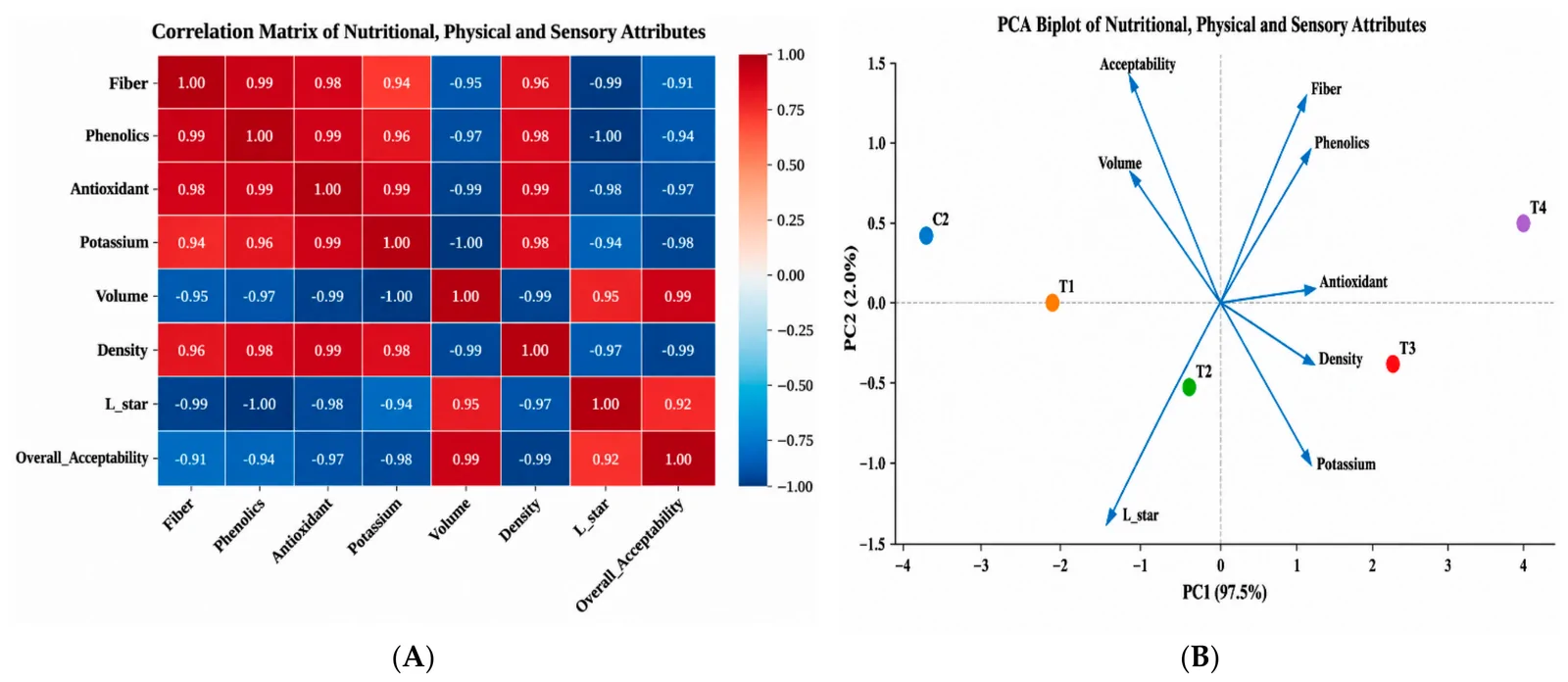
Multivariate analysis of nutritional, physical, color, and sensory characteristics of oat-based cookies fortified with doum fruit powder (DFP). (**A**) Pearson correlation matrix illustrating the relationships among selected nutritional, physical, color, and sensory attributes. Red and blue colors indicate positive and negative correlations, respectively, while the numerical values represent Pearson’s correlation coefficients (r). (**B**) Principal component analysis (PCA) biplot showing the distribution of cookie formulations (C2, T1–T4) according to the evaluated quality attributes. PC1 and PC2 explained 97.5% and 2.6% of the total variance, respectively.

**Table 1:** Ingredient composition of oat cookies fortified with doum fruit powder (DFP).

Ingredients (g)	C1	C2	T1	T2	T3	T4
Oat flour	100	100	95	90	85	80
Doum fruit powder	-	-	5	10	15	20
Refined sugar	50	-	-	-	-	-
Date paste	-	50	50	50	50	50
Shortening	50	50	50	50	50	50
Skim milk powder	10	10	10	10	10	10
Baking powder	2	2	2	2	2	2
Salt	0.2	0.2	0.2	0.2	0.2	0.2
Egg	30	30	30	30	30	30
Water	Variable	Variable	Variable	Variable	Variable	Variable

C1: Control cookies prepared with oat flour and refined sugar. C2: Control cookies prepared with oat flour and date paste. T1–T4: Cookies formulated with 5–20% doum fruit powder substitution levels and sweetened with date paste.

**Table 2:** Proximate composition and energy value of oat flour (OF) and doum fruit powder (DFP) on a dry weight basis.

Component	Unit	Oat Flour (OF)	Doum Fruit Powder (DFP)
Moisture	g/100 g	7.54 ± 0.12 ^b^	10.22 ± 0.01 ^a^
Ash	g/100 g	1.78 ± 0.16 ^b^	6.52 ± 0.09 ^a^
Crude protein	g/100 g	14.20 ± 0.22 ^a^	4.16 ± 0.13 ^b^
Crude fat	g/100 g	7.97 ± 0.11 ^a^	0.83 ± 0.31 ^b^
Crude fiber	g/100 g	9.84 ± 0.16 ^b^	15.43 ± 0.17 ^a^
Total carbohydrates	g/100 g	66.21 ± 0.38 ^b^	73.06 ± 0.18 ^a^
Energy	kcal/100 g	393.37 ± 1.23 ^a^	316.35 ± 0.56 ^b^

Values are expressed as mean ± standard deviation (SD) of three independent determinations (*n* = 3). Means within the same row followed by different superscript letters differ significantly at *p* ≤ 0.05 according to Duncan’s Multiple Range Test.

**Table 3:** Bioactive compounds and antioxidant activity of oat flour (OF) and doum fruit powder (DFP).

Bioactive Compound	Unit	Oat Flour (OF)	Doum Fruit Powder (DFP)
Total phenolic content (TPC)	mg GAE/100 g DW	86.34 ± 0.44 ^b^	128.32 ± 0.23 ^a^
Total flavonoid content (TFC)	mg QE/100 g DW	312.22 ± 0.56 ^a^	82.64 ± 0.45 ^b^
β-Carotene	mg/100 g DW *	12.32 ± 0.23 ^a^	9.31 ± 0.11 ^b^
DPPH radical scavenging activity	% inhibition	32.48 ± 0.12 ^b^	49.89 ± 0.76 ^a^

Values are expressed as mean ± SD (*n* = 3). Means within the same row followed by different superscript letters are significantly different at *p* ≤ 0.05 according to Duncan’s Multiple Range Test. * DW = dry weight; GAE = gallic acid equivalents; QE = quercetin equivalents.

**Table 4:** Mineral composition of oat flour (OF) and doum fruit powder (DFP).

Mineral	Unit	Oat Flour (OF)	Doum Fruit Powder (DFP)
Calcium (Ca)	mg/100 g	62.89 ± 0.14 ^b^	276.82 ± 1.10 ^a^
Magnesium (Mg)	mg/100 g	134.12 ± 0.67 ^b^	221.36 ± 2.05 ^a^
Potassium (K)	mg/100 g	481.56 ± 1.20 ^b^	2853.00 ± 4.22 ^a^
Sodium (Na)	mg/100 g	42.11 ± 0.11 ^b^	258.48 ± 1.23 ^a^
Zinc (Zn)	mg/100 g	1.22 ± 0.004 ^a^	1.06 ± 0.005 ^b^
Copper (Cu)	mg/100 g	1.98 ± 0.10 ^a^	0.43 ± 0.003 ^b^
Iron (Fe)	mg/100 g	3.08 ± 0.23 ^b^	6.26 ± 0.34 ^a^
Manganese (Mn)	mg/100 g	4.78 ± 0.45 ^a^	0.84 ± 0.23 ^b^
Phosphorus (P)	mg/100 g	401.56 ± 1.12 ^a^	158.88 ± 0.45 ^b^
Nickel (Ni)	mg/100 g	ND	0.018 ± 0.006 ^a^
Cobalt (Co)	mg/100 g	ND	0.007 ± 0.003 ^a^

Values are expressed as mean ± SD (*n* = 3). Means within the same row followed by different superscript letters differ significantly at *p* ≤ 0.05 according to Duncan’s Multiple Range Test. ND = Not detected.

**Table 5:** (**A**) HPLC profile of major phenolic compounds identified in oat flour (OF) and doum fruit powder (DFP). (**B**) HPLC profile of major phenolic compounds identified in oat flour (OF) and doum fruit powder (DFP).

**(A)**				
**No.**	**Phenolic Compounds**	**Oat Flour (OF)**		**t_R_** **Min.**
		**Concentration (mg/kg)**	**Percentage (%)**	
1	Gallic	13.4	3.46	5.63
2	Vanillic	21.02	5.42	6.78
3	Syringic	20.18	5.21	7.15
4	Sinapic	14.22	3.67	7.32
5	Caffeic	6.65	1.72	8.56
6	Protocatchuic	1.12	0.29	9.22
7	Chlorogenic	2.04	0.53	9.44
8	Epicatachin	1.18	0.30	9.65
9	Ferulic	162.12	41.83	9.84
10	ACoumaric	3.78	0.98	10.22
11	p-Coumaric	82.45	21.28	10.42
12	Iso-ferulic	6.8	1.75	10.72
13	3-hydroxy4-methoxy cinnamic acid	9.6	2.48	10.83
14	p-Caempferol	32.61	8.41	11.35
15	p-OH-benzoic	3.2	0.83	11.91
16	Cinnamic	7.16	1.85	12.15
The total		387.53	100%	
**(B)**				
**No.**	**Phenolic Compounds**	**Doum Fruit Powder (DFP)**		**t_R_** **Min.**
		**Concentration (mg/kg)**	**Percentage (%)**	
1	Catechein	42.68	3.15	6.32
2	3-oH tyrosol	38.11	2.82	7.12
3	Protocatchuic	102.34	7.56	7.45
4	Chlorogenic	28.12	2.08	8.32
5	Epicatachin	112.56	8.32	8.56
6	P-OH-benzoic	178.45	13.19	9.11
7	Caffeic	18.43	1.36	9.44
8	Vanillic	22.18	1.64	9.65
9	4-amino-benzoic	9.88	0.73	9.72
10	Caffeine	42.11	3.11	9.82
11	Ferulic	14.38	1.06	10.12
12	Iso-ferulic	32.15	2.38	10.32
13	E- vanillic	366.27	27.07	10.48
14	Reversetrol	6.48	0.48	11.65
15	Ellagic	220.44	16.30	11.87
16	Alpha-coumaric	8.23	0.61	12.34
17	p-coumaric	54.11	4.00	12.87
18	Salycilic	36.92	2.73	13.10
19	Coumarine	6.32	0.47	13.76
20	Cinnamic	8.54	0.63	14.22
21	3,4,5-methoxy cinnamic	4.12	0.30	14.22
The total		1352.82	100	

**Table 6:** Functional properties of oat flour (OF) and doum fruit powder (DFP).

Functional Property	Unit	Oat Flour (OF)	Doum Fruit Powder (DFP)
Water absorption capacity (WAC)	mL g^−1^	1.94 ± 0.33 ^b^	3.25 ± 0.19 ^a^
Oil absorption capacity (OAC)	mL g^−1^	2.18 ± 0.002 ^a^	1.34 ± 0.10 ^b^
Water holding capacity (WHC)	g g^−1^	2.09 ± 0.65 ^a^	1.10 ± 0.44 ^b^
Oil holding capacity (OHC)	g g^−1^	0.78 ± 0.009 ^b^	0.98 ± 0.10 ^a^
Emulsion capacity (EC)	%	1.92 ± 0.23 ^a^	1.62 ± 0.17 ^b^
Bulk density (BD)	g cm^−3^	0.83 ± 0.008 ^a^	0.82 ± 0.001 ^a^
Swelling capacity (SC)	%	5.32 ± 1.10 ^a^	3.41 ± 2.02 ^b^

Values are expressed as mean ± SD (*n* = 3). Means within the same row followed by different superscript letters differ significantly at *p* ≤ 0.05 according to Duncan’s Multiple Range Test.

**Table 7:** Chemical composition, bioactive compounds, and antioxidant activity of oat flour cookies fortified with different levels of doum fruit powder (DFP).

Parameter	Unit	C1 (100% Wheat)	C2 (100% Oat)	OF:DFP (95:5)	OF:DFP (90:10)	OF:DFP (85:15)	OF:DFP (80:20)
Moisture	%	4.78 ± 0.61 ^d^	5.76 ± 0.37 ^b^	4.62 ± 0.50 ^c^	5.63 ± 0.34 ^b^	5.98 ± 0.38 ^b^	6.72 ± 0.30 ^a^
Crude protein	%	17.63 ± 0.23 ^a^	17.81 ± 0.22 ^a^	16.44 ± 0.16 ^b^	15.87 ± 0.20 ^b^	14.17 ± 0.29 ^c^	13.76 ± 0.35 ^d^
Crude fat	%	21.42 ± 0.17 ^a^	21.22 ± 0.20 ^a^	20.06 ± 1.02 ^b^	19.12 ± 0.66 ^b^	18.44 ± 0.45 ^c^	16.72 ± 0.33 ^d^
Crude fiber	%	5.62 ± 0.23 ^e^	5.81 ± 0.81 ^d^	5.98 ± 0.33 ^d^	6.63 ± 0.11 ^c^	7.51 ± 0.56 ^b^	8.69 ± 0.29 ^a^
Ash	%	1.12 ± 0.28 ^e^	1.28 ± 0.18 ^d^	1.33 ± 0.21 ^d^	1.58 ± 0.38 ^c^	1.71 ± 0.12 ^b^	2.20 ± 0.05 ^a^
Total carbohydrates	%	54.21 ± 0.65 ^e^	53.88 ± 0.23 ^e^	56.19 ± 0.45 ^d^	56.80 ± 1.01 ^c^	58.17 ± 0.77 ^b^	58.63 ± 1.09 ^a^
Energy	kcal/100 g	480.14 ± 1.23 ^a^	477.74 ± 1.36 ^b^	471.06 ± 0.66 ^c^	462.76 ± 0.87 ^d^	455.32 ± 0.98 ^e^	440.04 ± 0.46 ^f^
Total phenolics	mg GAE/100 g	35.71 ± 0.55 ^f^	37.08 ± 0.36 ^e^	39.78 ± 0.12 ^d^	41.11 ± 0.72 ^c^	45.68 ± 1.02 ^b^	50.04 ± 1.10 ^a^
Total flavonoids	mg QE/100 g	187.66 ± 0.89 ^a^	191.34 ± 0.84 ^a^	156.12 ± 0.62 ^b^	140.66 ± 0.46 ^c^	123.27 ± 1.10 ^d^	111.56 ± 1.02 ^e^
β-Carotene	mg/100 g	6.72 ± 1.10 ^a^	7.22 ± 0.88 ^a^	6.45 ± 0.48 ^b^	5.78 ± 0.56 ^c^	5.02 ± 0.81 ^d^	4.77 ± 0.11 ^d^
DPPH radical scavenging activity	%	15.42 ± 0.08 ^e^	15.67 ± 0.12 ^e^	17.22 ± 0.62 ^d^	19.41 ± 0.37 ^c^	21.78 ± 0.44 ^b^	24.11 ± 0.89 ^a^

Values are expressed as mean ± SD (*n* = 3). Means within the same row followed by different superscript letters differ significantly at *p* ≤ 0.05 according to Duncan’s Multiple Range Test.

**Table 8:** Mineral composition of oat flour cookies fortified with different levels of doum fruit powder (DFP).

Mineral	Unit	C1	C2	OF:DFP (95:5)	OF:DFP (90:10)	OF:DFP (85:15)	OF:DFP (80:20)
Potassium (K)	mg/100 g	196.40 ± 0.11 ^f^	284.22 ± 0.09 ^e^	325.45 ± 0.12 ^d^	373.78 ± 0.18 ^c^	401.34 ± 0.22 ^b^	434.28 ± 0.31 ^a^
Phosphorus (P)	mg/100 g	167.70 ± 0.07 ^d^	178.56 ± 0.44 ^c^	198.32 ± 0.44 ^b^	201.45 ± 0.44 ^b^	226.98 ± 0.44 ^a^	239.22 ± 0.44 ^a^
Magnesium (Mg)	mg/100 g	26.22 ± 0.44 ^e^	47.22 ± 0.07 ^d^	72.12 ± 0.07 ^c^	83.22 ± 0.07 ^b^	98.12 ± 0.07 ^b^	101.67 ± 0.07 ^a^
Calcium (Ca)	mg/100 g	33.22 ± 0.07 ^f^	48.12 ± 0.44 ^e^	73.77 ± 0.44 ^d^	89.56 ± 0.44 ^c^	100.23 ± 0.44 ^b^	132.14 ± 0.44 ^a^
Sodium (Na)	mg/100 g	16.22 ± 0.44 ^f^	35.83 ± 0.38 ^e^	74.22 ± 0.10 ^d^	89.68 ± 0.16 ^c^	128.67 ± 0.41 ^b^	142.11 ± 0.23 ^a^

Different letters indicate significant differences among treatments according to Duncan’s multiple range test (*p* ≤ 0.05).

**Table 9:** Physical characteristics of oat-based cookies are fortified with different levels of doum fruit powder (DFP).

Parameter	Unit	C1 (100% Wheat)	C2 (100% Oat)	OF:DFP (95:5)	OF:DFP (90:10)	OF:DFP (85:15)	OF:DFP (80:20)
Weight	G	18.00 ± 0.11 ^a^	17.88 ± 0.09 ^a^	17.64 ± 0.12 ^a^	17.12 ± 0.18 ^b^	16.82 ± 0.22 ^c^	16.10 ± 0.31 ^d^
Diameter	Mm	64.20 ± 0.44 ^a^	64.37 ± 0.38 ^a^	61.80 ± 0.10 ^b^	59.52 ± 0.16 ^c^	57.82 ± 0.41 ^d^	56.63 ± 0.23 ^e^
Thickness	Mm	7.60 ± 0.46 ^a^	7.67 ± 0.51 ^a^	7.41 ± 0.40 ^b^	7.24 ± 0.29 ^c^	6.74 ± 0.08 ^d^	6.58 ± 0.02 ^e^
Spread ratio	–	8.44 ± 0.07 ^b^	8.45 ± 0.08 ^b^	8.35 ± 0.13 ^c^	8.41 ± 0.21 ^b^	8.58 ± 0.80 ^a^	8.60 ± 0.34 ^a^
Volume	cm^3^	24.58 ± 0.34 ^a^	24.51 ± 0.31 ^a^	22.21 ± 0.08 ^b^	20.13 ± 0.23 ^c^	17.95 ± 0.03 ^d^	16.46 ± 0.16 ^e^
Density	g cm^−3^	0.73 ± 0.001 ^e^	0.73 ± 0.010 ^e^	0.79 ± 0.006 ^d^	0.84 ± 0.018 ^c^	0.94 ± 0.023 ^b^	0.98 ± 0.012 ^a^

Values are expressed as mean ± SD (*n* = 3). Means within the same row followed by different superscript letters differ significantly at *p* ≤ 0.05 according to Duncan’s Multiple Range Test.

**Table 10:** Sensory evaluation of oat-based cookies fortified with different levels of doum fruit powder (DFP).

Sensory Attribute	C1 (100% Wheat)	C2 (100% Oat)	OF:DFP (95:5)	OF:DFP (90:10)	OF:DFP (85:15)	OF:DFP (80:20)
Appearance	9.30 ± 0.78 ^a^	8.80 ± 0.71 ^b^	8.60 ± 0.56 ^b^	8.20 ± 0.44 ^c^	7.52 ± 0.69 ^d^	7.57 ± 0.70 ^d^
Color	9.10 ± 0.82 ^a^	8.70 ± 0.76 ^b^	8.60 ± 0.71 ^b^	8.20 ± 0.56 ^c^	7.50 ± 0.50 ^d^	7.40 ± 0.51 ^d^
Odor	9.10 ± 1.02 ^a^	9.00 ± 1.12 ^a^	8.10 ± 0.56 ^b^	7.55 ± 0.38 ^c^	7.20 ± 0.22 ^d^	6.90 ± 0.32 ^d^
Taste	9.10 ± 1.32 ^a^	8.85 ± 0.56 ^b^	8.10 ± 1.12 ^c^	7.40 ± 0.78 ^d^	7.20 ± 0.22 ^d^	6.98 ± 0.45 ^e^
Texture	8.60 ± 0.65 ^a^	8.10 ± 0.33 ^b^	7.80 ± 0.54 ^c^	7.38 ± 0.12 ^d^	7.00 ± 0.58 ^e^	6.60 ± 0.22 ^f^
Overall acceptability	9.04 ± 0.84 ^a^	8.69 ± 0.51 ^b^	8.24 ± 0.34 ^c^	7.75 ± 0.56 ^d^	7.12 ± 0.12 ^e^	7.09 ± 0.11 ^e^

Values are expressed as mean ± SD (*n* = 10). Means within the same row followed by different superscript letters are significantly different according to Duncan’s Multiple Range Test (*p* ≤ 0.05).

## Data Availability

The original contributions presented in this study are included in the article. Further inquiries can be directed to the corresponding author.
